# Towards a unified theory of correlations in recurrent neural networks

**DOI:** 10.1186/1471-2202-12-S1-P73

**Published:** 2011-07-18

**Authors:** Moritz Helias, Tom Tetzlaff, Markus Diesmann

**Affiliations:** 1 RIKEN Brain Science Institute, Wako City, Japan; 2 Department of Mathematical Sciences and Technology, Norwegian University of Life Sciences, Ås, Norway; 3 Institute of Neuroscience and Medicine (INM-6), Computational and Systems Neuroscience, Research Center; 4 Brain and Neural Systems Team, RIKEN Computational Science Research Program, Wako City, Japan

## 

Recent theoretical progress elucidated, why in recurrent networks the magnitude of correlations is much smaller than expected considering the amount of common input received by pairs of neurons [1,2]. The net inhibitory feedback is the underlying reason for the suppression of correlated activity in inhibition dominated networks [2], explaining the earlier observation of the cancellation of excitatory and inhibitory fluctuations in the synaptic afferents to each neuron in the network [1].

In [2] Fokker-Planck theory is used to reduce the integrate-and-fire dynamics to an effective linear rate model. Averaging over neuronal populations leads to closed form expressions for the time integrated pairwise correlation.

A different line of theoretical work uses Hawkes theory to study correlations [3]. Recently this theory was extended to capture inhibitory coupling and delayed interaction [4]. The latter work exposes that the asymmetry of the cross-correlation function relating the spiking of excitatory and inhibitory neurons is due to the reverberation of the network caused by the spikes of the two neurons. In networks without time delay the asymmetry appears as a time lag of inhibition with respect to excitation [1].

The predicted correlation functions, however, deviate quantitatively from direct simulations of integrate-and-fire networks. In the current work we present a unification of both theories [2,4] aiming at a self-consistent description of time-lagged correlations in recurrent networks.

The new theory is governed by a convolution equation for the cross-correlation resulting from a linear perturbation expansion of the neuronal dynamics. The perturbative approach is similar to [2] and the convolution equation is structurally the same as in [3]. For the example of a balanced recurrent network we obtain self-sonsistent expressions of the population-averaged correlation structure in the asynchronous irregular regime [5]. Our results elucidate the interplay between the single spike train auto-covariance and the cross-covariance in the network.

The unification of firing rate approximations [2], point process models [3,6], and integrate-and-fire dynamics [5] is a step towards a coherent picture of the correlation structure of recurrent networks enabling the mapping of results from abstract models [1,6,7] to biologically more realistic ones.
